# Mucosal-Dominant Stevens-Johnson Syndrome Presenting as Hemorrhagic Glossitis in a Multimorbid Patient: A Diagnostic Challenge

**DOI:** 10.7759/cureus.82907

**Published:** 2025-04-24

**Authors:** Bhavik R Patel

**Affiliations:** 1 Medicine/Dermatology, Lincoln Memorial University DeBusk College of Osteopathic Medicine, Knoxville, USA

**Keywords:** drug-induced reaction, hemorrhagic glossitis, immunosuppressive therapy, mucosal-predominant sjs, oral mucositis, stevens-johnson syndrome

## Abstract

Stevens-Johnson syndrome (SJS) is a rare but potentially life-threatening mucocutaneous reaction, most often triggered by medications or infections. While classical SJS involves cutaneous and mucosal surfaces, atypical presentations with isolated mucosal involvement are rarely reported and frequently under-recognized. We present a diagnostically challenging case of mucosal-predominant SJS manifesting as severe hemorrhagic glossitis without cutaneous lesions in a 60-year-old male with multimorbidity, including recurrent strokes, coronary artery disease, chronic kidney disease, and type 2 diabetes mellitus. His condition progressed despite initial antimicrobial treatment but improved significantly with systemic corticosteroids. This case underscores the importance of considering mucosal SJS in the differential diagnosis of severe oral mucositis, particularly in multimorbid patients. It highlights the utility of immunosuppressive therapy when the diagnosis is suspected.

## Introduction

Stevens-Johnson syndrome (SJS) and toxic epidermal necrolysis (TEN) both represent severe cutaneous adverse reactions (SCARs) marked by diffuse keratinocyte apoptosis that results in mucocutaneous blistering and epidermal detachment. These conditions exist along a spectrum, primarily differentiated by the extent of skin detachment; SJS involves less than 10% of body surface area (BSA), TEN affects more than 30%, and SJS/TEN overlap lies in between [1,2]. SJS carries a mortality rate of 1-10%, while TEN exceeds 30% [3].

Drugs remain the leading cause of SJS, especially sulfonamides, anticonvulsants, non-steroidal anti-inflammatory drugs (NSAIDs), and allopurinol. Infections (e.g., *Mycoplasma pneumoniae*), vaccinations, and sometimes unidentified exposures also contribute. Genetic predispositions, such as human leukocyte antigen (HLA)-B*15:02 in carbamazepine-induced SJS among Han Chinese populations, further elevate risk [4-6].

More than 90% of SJS/TEN patients exhibit mucosal involvement, typically affecting oral, ocular, and genital sites. Rarely does isolated mucosal tissue involvement occur, often classified as atypical SJS or Fuchs syndrome [7]. Patients with underlying comorbid conditions such as diabetes mellitus, chronic kidney disease, or immunosuppression may display atypical or attenuated inflammatory responses that delay diagnosis and complicate the clinical course [8]. These individuals also experience worse outcomes due to impaired healing and overlapping systemic illnesses.

Clinicians often postpone or overlook the diagnosis when typical skin lesions are absent. Because early recognition and discontinuation of the culprit drug significantly reduce morbidity and mortality [9], they must maintain a high index of suspicion. In cases where mucosal lesions occur without skin involvement, clinicians often initially consider more common etiologies. The differential diagnosis for isolated mucositis includes infectious causes (e.g., oral candidiasis, herpes simplex virus (HSV)), autoimmune diseases (e.g., pemphigus vulgaris, Behçet’s disease, systemic lupus erythematosus), nutritional deficiencies (e.g., vitamin B12 or iron deficiency), and drug-induced mucosal injury. These overlapping clinical features can obscure the diagnosis, particularly in immunocompromised or multimorbid patients. As such, maintaining a broad differential is essential when evaluating severe or rapidly progressive mucosal disease.

In this report, we present an atypical case of mucosal-predominant SJS in a medically complex patient who had no visible skin lesions but developed worsening oral mucosal exfoliation, bleeding, and pain that failed to respond to antimicrobial therapy.

## Case presentation

A 60-year-old White male patient presented to the emergency department with increasing right-sided weakness, dizziness, chills, and several episodes of non-bloody emesis. He denied chest pain, dyspnea, or fever. His medical history included recurrent cerebrovascular accidents (CVAs) with residual right-sided weakness, coronary artery disease status post-coronary artery bypass grafting (CABG), atrial fibrillation on chronic anticoagulation, stage III chronic kidney disease (CKD), type 2 diabetes mellitus with peripheral neuropathy and chronic bilateral diabetic foot ulcers, hypertension, hyperlipidemia, anxiety, and depression.

Due to his chronic lower extremity wounds, immunocompromised state, borderline hypotension, and elevated inflammatory markers, the clinical team suspected sepsis at presentation. They initiated a complete sepsis workup and began empiric broad-spectrum antibiotics with intravenous vancomycin 125 mg daily, cefazolin 2 g IV every eight hours, and clindamycin 900 mg IV every eight hours. On hospital admission, they held warfarin 5 mg daily, heparin 25,000 units in 250 mL D5W at 2000 units/hr continuous IV, heparin 6000 units IV push every four hours, and clopidogrel 75 mg daily due to mucosal bleeding risk. Elevated troponin levels raised concern for a type 2 non-ST elevation myocardial infarction (NSTEMI) due to systemic illness and dehydration. However, ECG findings remained unremarkable, and cardiac enzymes trended downward with supportive care. His history of coronary artery disease and prior CABG further increased his risk for myocardial oxygen supply-demand mismatch.

He also triggered a stroke alert due to the worsening of his baseline right-sided weakness. Neurology evaluated the patient and ruled out a new infarct on imaging. Clinicians attributed his symptoms to systemic decompensation. His history of CVAs likely stemmed from chronic comorbidities, including atrial fibrillation, diabetes, and hypertension. A podiatric examination confirmed chronic plantar ulcers on both feet. A right foot wound culture grew methicillin-sensitive *Staphylococcus aureus *and Group C *Streptococcu*s, although MRI ruled out abscess or osteomyelitis.

On hospital day three, the patient developed progressive oral pain and glossitis. Examination revealed white plaques and mucosal irritation. The team initially diagnosed oral candidiasis and prescribed nystatin oral liquid 500,000 units every six hours, which they discontinued after one day due to lack of improvement. They also prescribed a compounded oral suspension of diphenhydramine-aluminum-magnesium hydroxide-lidocaine 10 mL every six hours, which was later suspended. The team administered hydromorphone 1 mg IV push every three hours for symptom relief and lorazepam 0.25 mg IV push every three hours as needed.

As symptoms worsened, they escalated antifungal therapy to micafungin 100 mg IV daily and initiated acyclovir orally, later switched to an intravenous route for presumed herpes simplex reactivation. Despite these measures, the patient’s oral symptoms rapidly worsened.

Given the severity of mucosal involvement, the team considered a broad differential diagnosis, including infectious etiologies (e.g., candidiasis, HSV), autoimmune disorders (e.g., pemphigus vulgaris, Behçet’s disease), and drug-induced mucositis, including SJS. Although initial treatment targeted candidiasis and viral reactivation, the patient failed to improve. His severe hemorrhagic glossitis, mucosal sloughing, and lack of cutaneous involvement most closely aligned with mucosal-predominant SJS, also known as Fuchs syndrome.

By hospital day five, he developed severe mucosal exfoliation, hemorrhagic glossitis, and active oral bleeding. He reported worsening pain radiating to his ears and head and described feeling as though “debris was choking him.” Examination revealed sloughing of the tongue and inner cheeks, ulceration, and inability to tolerate oral intake. The team transitioned acyclovir to IV, continued micafungin, and suspended piperacillin-tazobactam. They avoided fluconazole due to QT prolongation. Given the mucosal bleeding, they held all anticoagulation and antiplatelet agents.

On hospital day six, the team administered dexamethasone 8 mg IV once, followed by methylprednisolone 125 mg IV daily (pulse dosing). Dermatology and ENT consultants confirmed a working diagnosis of mucosal-dominant SJS, given the rapid mucosal deterioration without ocular or cutaneous involvement. The team discontinued vancomycin, cefazolin, and clindamycin, suspecting a drug-induced mucocutaneous reaction, and held other non-essential medications. Given the temporal association and known link to severe cutaneous adverse reactions, the team identified oral vancomycin as the most likely offending agent. However, piperacillin-tazobactam was also considered a potential contributor. Both were suspended to eliminate suspected triggers and minimize further mucosal injury.

Over the next 48-72 hours, the patient demonstrated significant improvement in mucosal healing, oral intake, and pain control. He resumed eating soft foods and drinking fluids. On hospital day eight, after observing clinical stabilization, the team discontinued corticosteroids and discussed reinitiating anticoagulation. Autoimmune testing, including antinuclear antibody (ANA) and HSV polymerase chain reaction (PCR), returned negative. The patient was discharged in stable condition with a working diagnosis of mucosal-predominant SJS and transferred for further evaluation and biopsy.

The key laboratory findings over the hospital course is given in Table 1.

**Table 1:** Key laboratory findings ESR: erythrocyte sedimentation rate; MSSA: methicillin-sensitive *Staphylococcus aureus;* PCR: polymerase chain reaction; HSV: herpes simplex virus; T2DM: type 2 diabetes mellitus; AKI: acute kidney injury; CKD: chronic kidney disease; N/A: not available

Parameter	Patient Value	Reference Range	Interpretation
WBC	21.2 ×10⁹/L	4.0–11.0 ×10⁹/L	Marked leukocytosis (infection/inflammation)
Hemoglobin	11 g/dL	13.5–17.5 g/dL (male)	Mild anemia
Platelets	396 ×10⁹/L	150–400 ×10⁹/L	Normal
Creatinine	1.63 mg/dL	0.7–1.3 mg/dL	AKI on CKD
Troponin I (peak)	343 ng/L	<14 ng/L	Significant elevation
Glucose	221 mg/dL	70–110 mg/dL	Hyperglycemia (T2DM)
HSV-1/2 PCR (oral)	Not detected	—	Negative
ESR	Elevated (exact N/A)	<20 mm/hour	Inflammatory state
Foot wound culture	MSSA + Group C Strep	—	Confirmed local infection

## Discussion

SJS and TEN represent a spectrum of rare, life-threatening mucocutaneous hypersensitivity reactions characterized by widespread keratinocyte apoptosis. While medications such as sulfonamides, allopurinol, and antiepileptics are the most frequently implicated triggers, infections and systemic inflammation may also initiate these conditions [1,2]. SJS typically presents with skin detachment affecting less than 10% of the body surface area, accompanied by mucosal erosions in over 90% of cases. However, a subset of patients present with mucosal involvement in the absence of skin lesions, a phenomenon referred to as Fuchs syndrome or mucosal-predominant SJS [3].

In the present case, a 60-year-old male patient with multimorbidity developed progressive oral ulcerations, hemorrhagic glossitis, and mucosal bleeding without ocular, genital, or cutaneous manifestations. These features are consistent with mucosal-dominant SJS, a subtype more commonly reported in pediatric populations, particularly in association with *Mycoplasma pneumoniae* infection [4,5]. However, cases in adults are less commonly described and often pose a diagnostic challenge. The absence of skin involvement may lead to misdiagnosis as oral candidiasis, HSV infection, aphthous stomatitis, or autoimmune mucositis (e.g., pemphigus vulgaris or Behçet’s disease) [7]. In the current case, the patient failed to respond to antifungal and antiviral therapy, prompting reconsideration of the diagnosis.

**Figure 1:**
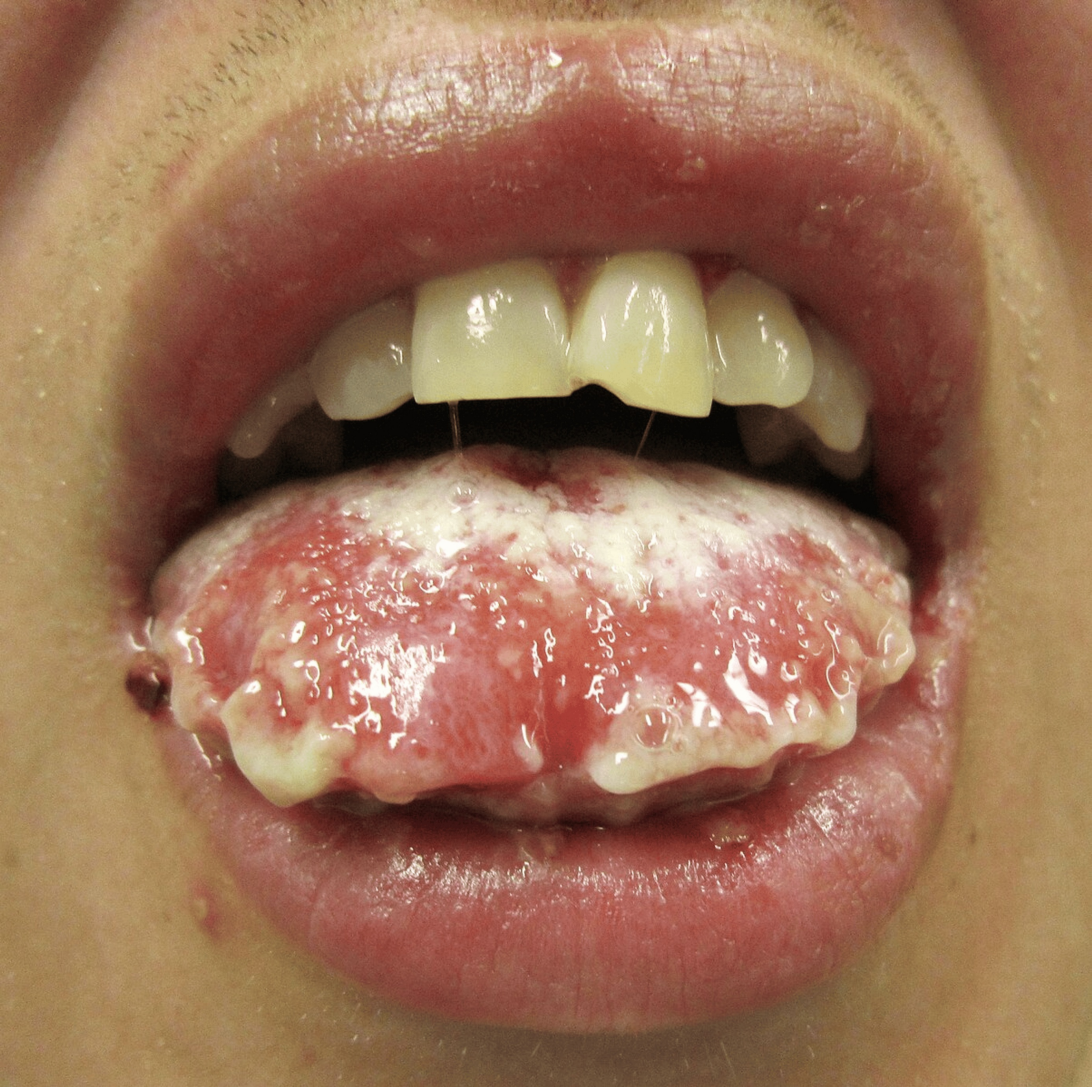
Representative image of oral mucosal desquamation in Stevens–Johnson syndrome. This clinical photograph shows extensive erythema, desquamation, and ulceration involving the lips and tongue. NOTE: The image is illustrative and not from the patient described in this report. Image Credit: James Heilman, MD. Licensed under Creative Commons (CC BY-SA 3.0), Attribution-ShareAlike 3.0 Unported. Source: Wikimedia Commons [10].

While histopathological confirmation was not obtained, the characteristic mucosal findings, unresponsiveness to standard treatment, and prompt clinical response to corticosteroids supported the diagnosis. A representative image of oral mucosal desquamation in SJS, illustrating findings similar to those seen in the current patient, is provided in Figure 1 [10].

Determining causality in SJS, especially in polypharmacy cases, can be complex. The ALDEN (Algorithm of Drug Causality in Epidermal Necrolysis) tool provides a structured framework that incorporates time to onset, known drug risk profiles, and dechallenge outcomes [9]. Although not formally applied in this case due to multiple chronic medications and no new exposures, the discontinuation of broad-spectrum antibiotics and anticoagulation coincided with the patient’s improvement, suggesting a potential role in either triggering or exacerbating the mucosal reaction.

The use of systemic corticosteroids in SJS/TEN remains controversial. Historically, their use was discouraged due to concerns regarding immunosuppression and infection risk. However, several recent studies have supported their use when initiated early, particularly in limited or mucosal-predominant disease [11]. In our case, the patient’s rapid clinical improvement within 72 hours of high-dose IV methylprednisolone supports emerging literature suggesting corticosteroids may attenuate immune-mediated epithelial injury and hasten recovery. Chang et al. emphasized the importance of early intervention, highlighting corticosteroids as a first-line immunomodulatory therapy during the apoptotic phase of SJS/TEN, when damage may still be reversible [12].

This case also illustrates the diagnostic and therapeutic complexity of atypical SJS in a multimorbid patient. Chronic illnesses such as diabetes and CKD can dampen or distort immune responses, mask classical features, and delay diagnosis. Early interdisciplinary collaboration, including dermatology, otolaryngology, and infectious disease, was essential in reaching the diagnosis and guiding appropriate therapy.

Ultimately, this case adds to the limited literature on mucosal-predominant SJS in adults. Recognition of this underreported presentation is essential to avoid delays in diagnosis and to initiate timely treatment. Clinicians should maintain a high index of suspicion for SJS in patients presenting with severe mucosal injury unresponsive to conventional antimicrobial or antiviral therapy, even in the absence of cutaneous signs.

## Conclusions

This case highlights a rare mucosal-predominant variant of SJS in an adult with multiple comorbidities, in which the absence of skin involvement delayed diagnosis and led to initial mismanagement. Rapid improvement following high-dose corticosteroid therapy supports growing evidence for their role in select cases, particularly where systemic involvement is limited. The case emphasizes the importance of recognizing atypical SJS presentations, utilizing interdisciplinary care, and applying structured tools like the ALDEN algorithm to guide diagnosis and management. Though literature on adult mucosal-only SJS remains limited, increased awareness may promote earlier recognition, reduce complications, and improve outcomes. Further studies are needed to define diagnostic criteria and optimal treatment strategies.
